# Advances in the therapeutic application and pharmacological properties of kinsenoside against inflammation and oxidative stress-induced disorders

**DOI:** 10.3389/fphar.2022.1009550

**Published:** 2022-10-04

**Authors:** Li Lu, Yuan Xiong, Ze Lin, Xiangyu Chu, Adriana C. Panayi, Yiqiang Hu, Juan Zhou, Bobin Mi, Guohui Liu

**Affiliations:** ^1^ Department of Orthopedics, Union Hospital, Tongji Medical College, Huazhong University of Science and Technology, Wuhan, China; ^2^ Hubei Province Key Laboratory of Oral and Maxillofacial Development and Regeneration, Wuhan, China; ^3^ Division of Plastic Surgery, Brigham and Women’s Hospital, Harvard Medical School, Boston, MA, United States; ^4^ Department of Hand-, Plastic and Reconstructive Surgery, Microsurgery, Burn Trauma Center, BG Trauma Center Ludwigshafen, University of Heidelberg, Ludwigshafen, Germany; ^5^ Department of Cardiology, Hubei Provincial Hospital of Traditional Chinese Medicine, Wuhan, China

**Keywords:** kinsenoside, herbal medicine, inflammation, oxidative stress, signal pathway, cytokine, ROS

## Abstract

Extensive research has implicated inflammation and oxidative stress in the development of multiple diseases, such as diabetes, hepatitis, and arthritis. Kinsenoside (KD), a bioactive glycoside component extracted from the medicinal plant *Anoectochilus roxburghii*, has been shown to exhibit potent anti-inflammatory and anti-oxidative abilities. In this review, we summarize multiple effects of KD, including hepatoprotection, pro-osteogenesis, anti-hyperglycemia, vascular protection, immune regulation, vision protection, and infection inhibition, which are partly responsible for suppressing inflammation signaling and oxidative stress. The protective action of KD against dysfunctional lipid metabolism is also associated with limiting inflammatory signals, due to the crosstalk between inflammation and lipid metabolism. Ferroptosis, a process involved in both inflammation and oxidative damage, is potentially regulated by KD. In addition, we discuss the physicochemical properties and pharmacokinetic profiles of KD. Advances in cultivation and artificial synthesis techniques are promising evidence that the shortage in raw materials required for KD production can be overcome. In addition, novel drug delivery systems can improve the *in vivo* rapid clearance and poor bioavailability of KD. In this integrated review, we aim to offer novel insights into the molecular mechanisms underlying the therapeutic role of KD and lay solid foundations for the utilization of KD in clinical practice.

## Introduction

Inflammation is orchestrated by enhanced vessel dilation and capillary permeability, accompanied by the recruitment of immune cells, such as neutrophils, monocytes, and lymphocytes, and it has been described as a vast array of biological processes that function together to protect the host against insults of external and internal pathogenic factors, including microbial infection, traumatic injury, and autoantibody attack (Yeung et al., 2018; Medzhitov, 2021). Overactivation of this defense system, induced by excessive inflammation and the overproduction of pro-inflammatory substances can be detrimental for the rehabilitation of the structural and functional integrity of the body tissues and can even result in a life-threatening cytokine storm and systemic inflammation response syndrome (Yu et al., 2020; Panigrahy et al., 2021). This toxic inflammation results in the pathological events implicated in the development of various diseases, such as atherosclerosis, myocarditis, arthritis, osteomyelitis, and cancer. The resolution of inflammation has been proven to be a feasible strategy for illness amelioration (Liu Y. X. et al., 2022; Komatsu and Takayanagi, 2022; Kong et al., 2022; Rausch et al., 2022; Tan et al., 2022).

There is evidence that reactive oxygen species (ROS), mainly superoxide anion, hydrogen peroxide, and hydroxyl radicals, serve as secondary messengers applied for transmitting intracellular signals. In this context, ROS plays a role in a wide range of physiological processes. Oxidative stress occurs when processes, such as ischemia, hypoxia, and toxicosis, shift the balance towards ROS generation, and ROS production exceeds clearance, leading to a disturbance in redox balance and uncontrolled ROS accumulation (Dunnill et al., 2017; Shaito et al., 2022). Oxidative stress induces lipid peroxidation, protein denaturation, and DNA breakage, which, in turn, triggers the dysregulation of cellular homeostasis and contribute to disorders progression (Peng et al., 2022). The literature has shown that oxidative stress is involved in fracture non-union, diabetic ulcers, intervertebral disc degeneration, and ischemic reperfusion damage, suggesting that agents capable of decreasing ROS content may be therapeutic (Baek and Lee, 2016; Muinos-Lopez et al., 2016; Wang et al., 2019; Jiang et al., 2021).

**FIGURE 1:**
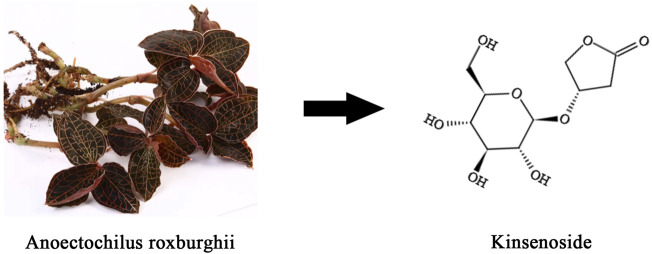
The whole plant of *Anoectochilus roxburghii* and the chemical structural formula of KD.

Herbal products with varying pharmacological activities have been widely used for managing disease and improving health across Asia and particularly in China (Shi et al., 2022). *Anoectochilus roxburghii* (Wall.) Lindl. (*A. roxburghii*), which belongs to the genus *Anoectochilus* and the Orchidaceae family, is a perennial ethnomedicinal plant widely distributed in tropical and subtropical regions (Ye et al., 2017). Kinsenoside (KD) is the principle bioactive constituent isolated from *A. roxburghii*, and its therapeutic effects have been identified in multiple diseases, including diabetes, hepatitis, osteoarthritis, dyslipidemia, osteoporosis, acute lung injury, and endotoxin shock (Figure 1). In particular, KD is reported to display potent anti-inflammatory abilities *via* suppressing the activation of certain immunocytes and the production of pro-inflammatory cytokines, as well as promoting the synthesis and release of anti-inflammatory factors (Qi et al., 2018). Meanwhile, KD is capable of inhibiting the progression of oxidative stress by decreasing the activities of ROS-producing enzymes, such as nicotinamide adenine dinucleotide phosphate (NAPDH) oxidases (NOXs), and enhancing the levels of ROS-scavenging antioxidants, such as superoxide dismutase (SOD), glutathione (GSH), and catalase (CAT). Given that the inflammatory response and oxidative stress are involved in multiple pathophysiological processes, this review seeks to summarize and evaluate the therapeutic functions of KD from the perspective of alleviating inflammation and oxidative stress, hoping to provide novel insights into the molecular mechanisms underlying KD’s ability to limit pathological processes and ultimately pave the way for the application of KD as a promising drug candidate in future clinical practice.

## Potentials of KD against disease development

Both *in vitro* and *in vivo* studies have suggested that the initiation and progression of inflammation involve numerous complex interrelated processes, among which are the signal transduction pathways of mitogen-activated protein kinases (MAPKs), toll-like receptor 4 (TLR4), phosphatidylinositol 3-kinase (PI3K)/protein kinase B (Akt), janus tyrosine kinase (JAK)/signal transducer and activator of transcriptions (STATs), and nuclear factor kappa-B (NF-κB), which present the highest connection (Yeung et al., 2018; Yu et al., 2020; Decout et al., 2021). As previously reported, ROS are mainly synthesized by cytosol NOXs and xanthine oxidase. Moreover, the mitochondrial respiratory chain is identified as another important source of ROS generation through electron delivery and oxidative phosphorylation (Dunnill et al., 2017; Shaito et al., 2022). To maintain the dynamic equilibrium of the redox state, antioxidants including SOD, glutathione peroxidase (GPX), NADPH quinone oxidoreductase 1 (NQO1), CAT, and heme oxygenase 1 (HO-1) are produced to counteract superfluous ROS (Arulselvan et al., 2016). The detailed anti-inflammatory and anti-oxidative actions of KD for alleviating disease development are depicted in Table 1.

**TABLE 1:** Basic information on KD-related treatment objects and modalities.

Object	Stimulation	Animal model	Route of KD	Dosage of KD	Intervention time	References
ICR mice	CCl4	Chronic hepatitis	Oral gavage	17.5–270 mg/kg	3–8 w	Wu et al. (2007)
ICR mice	CCl4	Hepatic fibrosis	Oral gavage	50–150 mg/kg	3 w	Hsieh et al. (2011)
BALB/c mice	TAA	Hepatic fibrosis	Oral gavage	36–180 mg/kg	12 w	Wu et al. (2010)
C57BL/6 mice	DEN	Hepatic damage	Intraperitoneal injection	5 mg/kg	4 w	Yijie Sun et al. (2022)
C57BL/6J mice	Ethanol	Acute alcoholic liver injury	Oral gavage	10–40 mg/kg	31 days	Zou et al. (2019)
C57BL/6J mice	Ethanol, CCl4	Alcoholic liver injury	Oral gavage	20–40 mg/kg	9 w	Gao et al. (2021)
SD rats	EE	Cholestatic liver injury	Oral gavage	50–200 mg/kg	10 days	Ming et al. (2021)
C57BL/6 mice	ConA	Autoimmune hepatitis	Oral gavage	10–30 mg/kg	3–5 days	Xiang et al. (2016)
C57BL/6J mice	CCl4	Hepatic fibrosis	Oral gavage	10–30 mg/kg	8 w	Xiang et al. (2022)
Wistar rats	STZ	Diabetes	Oral gavage	5–15 mg/kg	21 days	Zhang et al. (2007)
ICR mice	STZ	Diabetes	Oral gavage	50–100 mg/kg	21 days	Liu et al. (2013)
C57BL/6J mice	ACLT	Osteoarthritis	Intraperitoneal injection	2.5–10 mg/kg	4 w	Zhou et al. (2019)
SD rats	MSU crystals	Gouty arthritis	Intra-articular injection	2.5–10 mg/kg	3 days	Han et al. (2016)
DBA/1 J mice	Type II collagen	Rheumatoid arthritis	Oral gavage	100–300 mg/kg	21 days	Hsiao et al. (2016)
ICR mice	OVX	Osteoporosis	Oral gavage	100–300 mg/kg	4 w	Hsiao et al. (2013)
SD rats	Puncture	IDD	Intraperitoneal injection	10 mg/kg	4 w	Wang et al. (2019)
ICR mice	LPS	Endotoxic shock	Intraperitoneal injection	100–300 mg/kg	3 days	Hsiao et al. (2011)
C57BL/6 mice	LPS	Acute lung injury	Oral gavage	100 mg/kg	7 days	Yue Yang et al. (2022)
C57BL/6 mice	HFD	Hyperlipemia	Oral gavage	50–100 mg/kg	2 w	Lee et al. (2019)

CCl4, carbon tetrachloride; TAA, thioacetamide; DEN, diethylnitrosamine; EE, 17α-ethinylestradiol; ConA, concanavalin A; STZ, streptozotocin; ACLT, anterior cruciate ligament transection; MSU, monosodium urate; OVX, ovariectomized; LPS, lipopolysaccharide; HFD, high fat diet; IDD, intervertebral disc degeneration.

### Hepatic disorders

**FIGURE 2:**
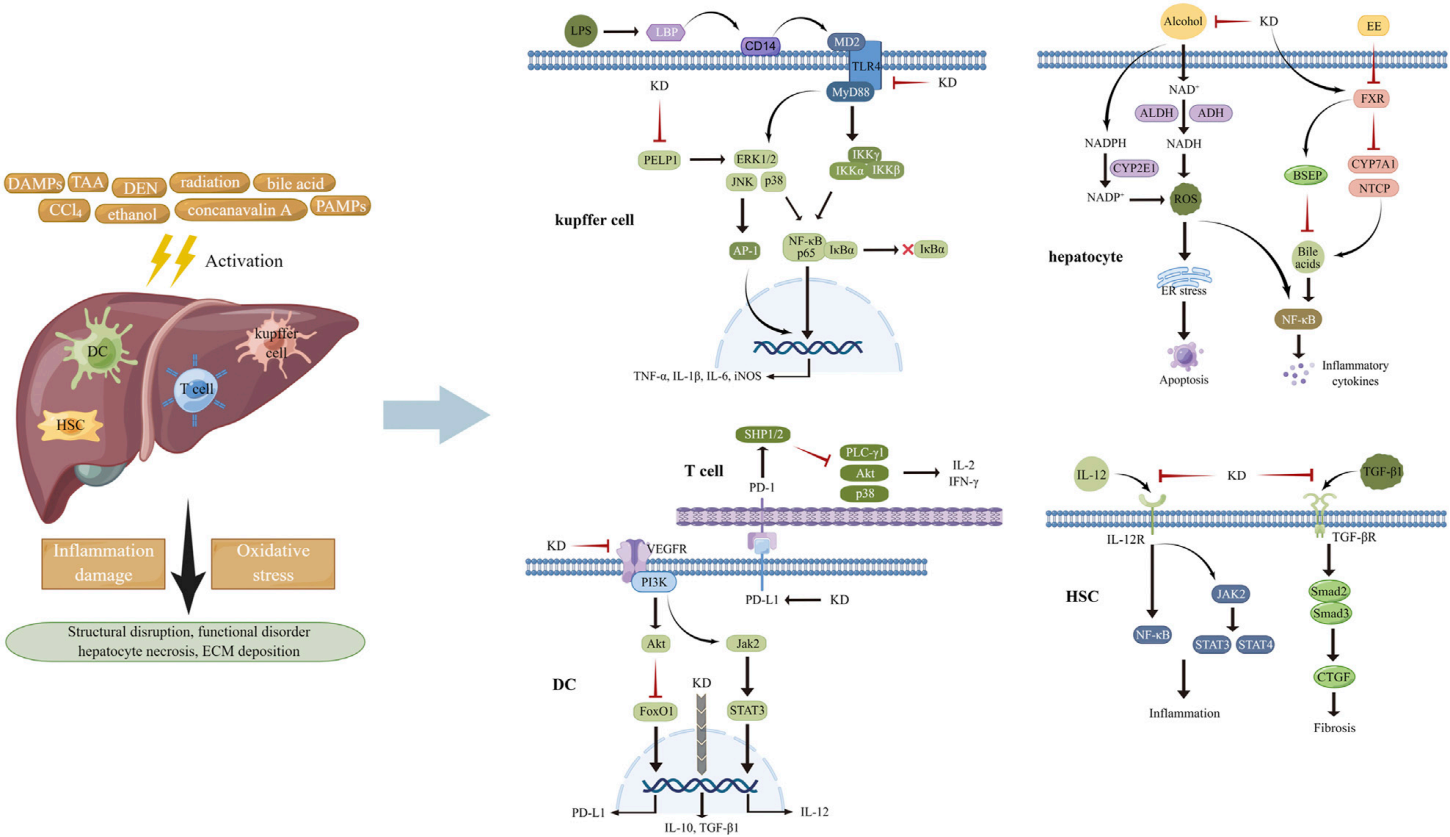
Various stimuli provoked inflammation response and oxidative stress in multiple cell types of liver tissues, attributed to activating MAPKs, STATs, NF-κB, and AP-1 and increasing the generation of ROS. KD was capable of impeding pro-inflammatory signaling pathways and suppressing the activities of ROS-producing enzymes, thereby improving subsequent structural disarrangement and functional dysregulation.

When exposed to hazardous compounds such as chemicals and environmental toxins, hepatic cells, especially Kupffer cells which are the resident macrophages in the liver, become activated and release various bioactive factors including pro-inflammatory cytokines and ROS, initiating the defense response for tissue repair (Marin et al., 2020; Matsubara et al., 2022). However, if the extent of inflammation reaction and oxidative stress outweighs the scavenging capacity of the host, injury to affected area occurs (Li et al., 2015; Yang Y. M. et al., 2022). Oral administration of carbon tetrachloride (CCl_4_) in mice elicited the appearance of hepatitis with elevated levels of liver hydroxyproline, increased spleen weight, and blood glutamic pyruvic transaminase activity and a lowered content of circulating albumin. These changes could be reversed by KD-carrying extracts from *Anoectochilus formosanus*, partly likely to be KD-related alleviation of hepatic damage triggered by hydrogen peroxide (H_2_O_2_) (Wu et al., 2007). Due to the validity and scope limitation of contemporary remedies, investigations have been undertaken for deciphering the hepatoprotective mechanisms of KD (Figure 2).

After entering into the body, lipopolysaccharide (LPS) from microbial pathogens is recognized by LPS-binding protein (LBP) and transported to CD14, which launches the trafficking of this foreign glycolipid to TLR4-myeloid differentiation protein 2 (MD2) heterodimers. The interaction of LPS with TLR4-MD2 transmits inflammatory signals to downstream transcription factors like NF-κB and interferon regulator factor 3, finally resulting in inflammation propagation (Bhatelia et al., 2014; Braza et al., 2016). MAPKs, including extracellular regulated protein kinase (ERK), p38 and c-Jun N-terminal kinase (JNK), play vital roles in inflammation mediation *via* regulating the activation of NF-κB, activator protein 1 (AP-1), and STATs through three-tiered, sequentially acting kinase cascades, in response to physical and chemical stimuli (Cargnello and Roux, 2011; Kassouf and Sumara, 2020). During the canonical NF-κB pathway activation, an enzyme complex containing catalytic subunits IκB kinase α (IKKα) and IKKβ and modulator IKKγ phosphorylates proteins of IκB family which undergo ubiquitinated degradation. Then NF-κB (p65-p50 dimer) is released and translocates to the nucleus for binding to the promoter of inflammatory genes, including interleukin (IL)-1β, IL-6, and tumor necrosis factor α (TNF-α) and initiating their transcription, ultimately causing the damage of target tissues (Hoesel and Schmid, 2013; Sun, 2017; Cai and Lin, 2022). A considerable amount of data indicated that limiting the activities of NF-κB and its upstream signal components helped mitigate the development of inflammation (Yu et al., 2020). According to the findings from Hsieh et al. (2011), KD treatment repressed the increase of alanine aminotransferase (ALT) and aspartate aminotransferase (AST) activities and hepatocellular necrosis in mice damaged by CCl4. Mechanistic analyses *in vivo* and *in vitro* had suggested that the inhibitory effects of this botanical derivative on the inflammation pathways of macrophages and hepatic tissues might underlie its actions in liver protection, as determined by decreased levels of CD14 and phosphorylated ERK1/2, p38 and JNK, followed by reduced phosphorylation of IκBα and transduction suppression of NF-κB/inducible nitric oxide synthase (iNOS)/nitric oxide (NO). This was done in agreement with Wu and colleagues, who showed that administration of KD repressed the LBP/CD14/TLR4 and TNF receptor signaling cascade to delay inflammation activation in hepatocytes and improve thioacetamide-induced liver injury (Wu et al., 2010). Moreover, another study, performed by Sun Y. et al. (2022), showed that intraperitoneal injection of KD markedly ameliorated diethylnitrosamine-triggered content increase in serum ALT, AST, and alkaline phosphatase (ALP) and destruction of liver morphology and cellular structure by blocking inflammatory pathways, including proline glutamic acid and leucine rich protein 1/ERK axis, thus decreasing the production of pro-inflammatory cytokines, such as TNF-α, IL-1β, and IL-6 and the associated concomitant impairments.

After its absorption in the blood and transportation to the hepatocytes, ethanol is primarily converted to acetaldehyde by alcohol dehydrogenase (ADH). Acetaldehyde is then oxidized to acetate by acetaldehyde dehydrogenase (ALDH). Catalytic courses of ADH and ALDH utilize nicotinamide adenine dinucleotide (NAD^+^) as an intermediate carrier of electrons, which evokes a decline in the ratio of NAD^+^ to NADH, thereby favoring ROS generation and depleting the cellular antioxidant reserves (Li et al., 2015). Cytochrome P-450 2E1 (CYP2E1)-mediated alcohol conversion is found to be a pathway that produces ROS in bulk. Dysfunction of the mitochondrial respiratory chain and activation of NOXs provoked by alcoholic stimulation are other sources of ROS synthesis (Rumgay et al., 2021; Li et al., 2022). The widespread presence of ROS induces the degeneration of constitutive proteins to activate endoplasmic reticulum (ER) stress and following cellular apoptosis. Increased malondialdehyde (MDA) and decreased in GSH have been noted in hepatic tissues of mice experiencing acute liver injury caused by alcohol intake (Li et al., 2015; Yang Y. M. et al., 2022). KD administration significantly lowered the level of blood AST and ALT, improved the structural disarrangement of tissue, alleviated cellular edema, decreased the formation of fat vacuoles and ballooning denaturation, and restored the abnormal trend of MDA and GSH in the alcoholic hepatic damage model. The mechanism of the actions underlying the liver-protecting effects of KD could be the expression inhibition of CYP2E1 and the enhancement of antioxidants synthesis, accompanied by ROS content reduction and oxidative stress suppression (Zou et al., 2019). Similarly, long-term ethanol consumption raised the content of serum AST and ALT, enhanced ballooning degeneration and hepatocyte apoptosis, and elevated the level of Bcl-XL and diminished the level of Bax in the hepatic tissues of mice, while these changes were counteracted with KD treatment. By weakening ethanol metabolism-elicited oxidative stress and subsequent ER stress and inflammation, KD played favorable roles against liver impairment, as indicated by a decline in ADH activity, CYP2E1 content and 3-nitrotyrosine and 4-hydroxynonenal expression, and elevation of CAT and SOD activity and the ratio of GSH/oxidized glutathione and NAD^+^/NADH. Other functions include inhibition of F4/80 and CD3 expression and inflammatory cell infiltration, reduction of BIP and CHOP and of eIF2α and JNK phosphorylation in the liver tissue, along with a decrease in TNF-α and IL-6 concentration in the circulation (Gao et al., 2021).

It should be noted that alcohol drinking restrains the expression of hepcidin that is implicated in the elimination of the ferroprotein participating in duodenal iron absorption, thus leading to iron overload, a hallmark of the pathogenesis of alcoholic liver disease (ALD) (Zhou X. et al., 2022). This phenomenon, along with the fact that ethanol strengthens transferrin receptor expression to promote iron uptake in the liver, implies that hepatic iron is elevated in response to alcohol use. This potentiates oxidative stress by increasing ROS generation through the Fenton reaction and iron-containing enzymes and then causes injury of the membrane lipids to induce inflammatory activation of Kupffer cells and ferroptosis, that is, iron-dependent programmed cell death (Li et al., 2022). It has been demonstrated that ferroptosis displays an accelerative role in promoting the development of various diseases, including hepatitis, liver fibrosis, ischemia reperfusion injury, stroke, cardiac hypertrophy, and cancers (Li et al., 2020; Chen et al., 2021; Tang et al., 2021). In addition to the Fenton reaction, several pathways have been identified to participate in ferroptosis-related ROS generation and lipid peroxidation, such as cystine/glutamate antiporter/GPX4, polyunsaturated fatty acid/lipoxygenase, p53/spermidine/spermine N^1^-acetyltransferase 1, and ferroptosis suppressor protein 1/coenzyme Q10 axis (Li et al., 2020; Chen et al., 2021). It has also been indicated that accumulation of excessive inflammatory cytokines and aberration of regulatory roles from immune cells are revealed during the process of ferroptosis (Wang and Lu, 2022). Therefore, it is possible that KD performs hepatoprotective action in ethanol abuse by disrupting the development of ferroptosis through mechanisms involving oxidative stress and inflammation regulation, which is elusive and requires further exploration.

It has been demonstrated that excessive bile acids (Bas) induce mitochondrial dysfunction to heighten ROS production, which, in turn, causes oxidative damage to organelle membranes and genetic materials, thus facilitating hepatocyte death (Gossard and Talwalkar, 2014). Bas also interact with the receptors to stimulate activities of inflammatory signal factors including NF-κB, MAPKs, Akt, and NLRP3 and fulfill hepatic necroptosis and inflammation-related impairment. Among the enzymes governing the metabolism of Bas, farnesoid X receptor (FXR) is a key element that controls biliary gathering by up-regulating proteins involved in scavenging and export and down-regulating proteins involved in generation and import of BA (Bertolini et al., 2022; Jiao et al., 2022; Trauner and Fuchs, 2022). As depicted in histological data, liver tissue of 17α-ethinylestradiol (EE)-stimulated rats was subjected to increased inflammatory cell infiltration, bile duct cell proliferation, hepatocellular oedema and necrosis, decreased distribution of perinuclear ER and mitochondria, and severe deficiency of cytoplasm, elucidating the successful establishment of an animal model of cholestasis. However, KD treatment dramatically inhibited the increase of liver body index, accelerated the decrease of biochemical indicators reflecting liver function damage and cholestatic impairment, and reversed the hepatic histopathological changes in rates with the subcutaneous injection of EE. KD-induced inhibition of the activation of pro-inflammatory NF-κB axis and the expression of downstream IL-1β and IL-6 could be implicated in the mechanisms of EE-insulted hepatic injury alleviation (Ming et al., 2021). Apart from directly antagonizing inflammation, KD elevated FXR content in hepatocytes to enhance the expression of bile salt export pump and repress the expression of Na^+^-dependent taurocholate cotransport peptide and cytochrome P450 7A1, resulting in amelioration of BA overload, an initiator of inflammation cascades, thereby indirectly limiting inflammatory signaling activation and effector-provoked liver injury.

Dendritic cells (DCs), a type of highly efficient antigen-presenting cells, are intimately associated with the activation of the adaptive immune response and acquisition of tolerogenic phenotype (Morante-Palacios et al., 2021). Upon exposure to pathological stimuli such as DNA and RNA from apoptotic cells and anti-microbial neutrophil extracellular traps, DCs capture and process these self-antigens and then transfer them to draining lymph nodes, where they are presented to T cells triggering their activation through combination of antigen peptide-MHC class I/II complex and co-stimulatory molecules with relevant receptors. Activated T cells secrete cytotoxic perforin/granzyme and pro-inflammatory cytokines to obliterate the source of self-antigens and lead to tissue damage (Sozzani et al., 2017; Worbs et al., 2017; Tiberio et al., 2018; Fousert et al., 2020). As previously reported, T cells are activated to induce autoimmune hepatitis (AIH) in mice insulted by concanavalin A, with increased contents of ALT and AST in the serum and severe histopathological injury, substantial lymphocyte infiltration, and elevated proportion of CD8^+^ T cells in the liver. After administration of KD, the abnormal manifestations were markedly improved, and the contents of interferon (IFN)-γ, IL-2, NO and MDA were diminished and concomitantly levels of transforming growth factor (TGF)-β1, IL-10, SOD and T_regs_ were increased, hinting that mitigating of inflammation and oxidative stress was involved in the therapeutic roles of KD. For elucidating its anti-inflammatory mechanisms, KD was observed to induce immune tolerance *via* up-regulation of co-inhibitory molecules PD-1/PD-L1, which hindered DC-mediated cross-priming of CD8^+^ T cells, thus disrupting the latter’s overactivation. In addition, given that immature DCs possessed lower capability for antigen-presentation and expression of co-stimulatory molecules and promoted clonal deletion, clonal anergy, and T_regs_ differentiation, KD was likely to confer DCs vital roles in peripheral tolerance through not only by impeding inflammation reactions but also disrupting DCs maturation, as seen by level reduction of IL-12, MHC-II, and CD86 and level increase of IL-10, TGF-β1, indoleamine-2,3-dioxygenase and immunoglobulin-like transcript 3. In terms of the pro-inflammatory signals targeted by KD, signaling transduction between the JAK2/STAT3 cascade and vascular endothelial growth factor receptor 2 (VEGFR2)/PI3K/Akt pathway was described as an important factor contributing to the hepatic damage caused by DCs and CD8^+^ T cells (Xiang et al., 2016). Moreover, due to the pathogenic actions of ROS overload in liver diseases, deciphering the anti-oxidative actions of KD will provide novel insight to the application of this compound in AIH treatment.

During the liver damage, HSCs in a resting condition are stimulated by fibrogenic mediators such as cytokines and are transformed to activated myofibroblasts, which produce and secrete excessive fibrosis-related proteins such as collagen and fibronectin to the perisinusoidal space, thereby leading to distortion of tissue structure and hepatic dysfunction (Wu et al., 2007; Wu et al., 2010; Hsieh et al., 2011; Gao et al., 2021; Nie et al., 2022). Since a series of preclinical data have confirmed the positive roles of the inflammatory response and oxidative stress in exacerbating liver fibrosis, strategies impeding inflammatory cascades or relieving ROS burden display excellent anti-fibrotic abilities (Binatti et al., 2022; Nassir, 2022). In a study performed by Xiang et al. (2022), CCl4 injection induced lymphocyte accumulation and collagen deposition in the inflammatory-rich milieu of hepatic tissues, KD administration lowered the generation of pro-inflammatory factors. including IL-2, IFN-γ, TNF-α, NO, and IL-12 and fibrosis-related proteins such as α-smooth muscle actin, TGF-β1, collagen type I, tissue inhibitors of metalloproteinases (TIMP)-1, and TIMP-2 and raised the release of anti-inflammatory cytokine IL-10 and extracellular matrix (ECM)-degrading MMP-13. In light of the pro-fibrotic behaviors of DCs in hyperinflammatory environments, Xiang and collaborators discovered that the suppression produced by KD on DCs-induced fibrogenesis was partly attributed to decreasing the activation of PI3K/Akt/FoxO1 axis, followed by an expression elevation of PD-L1, secretion reduction of IL-12, and immature phenotype polarization, then invalidating cytokines and immune cells-triggered HSCs transdifferentiation to myofibroblasts. Furthermore, Masson’s trichrome staining showed that KD lowered the content of collagen fibers in the liver exposed to alcohol *via* an avenue relying on enhancing ROS scavenging and repressing IL-6 and TNF-α production. Additionally, the switching of M1 macrophages to the M2 phenotype and inactivation of TLR4 signal pathway was implicated in the anti-fibrotic effects of KD. Interestingly, TGF-β1 was described as an inflammation-inhibiting factor and was up-regulated by KD in the model of autoimmune hepatitis, while it was reported that KD limited the expression of TGF-β1 to mitigate the development of fibrosis in the damaged liver (Xiang et al., 2016; Nie et al., 2022). Several factors, including animal model type, inductive agent, stimulation dose and duration, and disorder stage might explain this paradox.

### Dysglycemia-related injury

It is widely held that diabetes mellitus (DM) is one of the leading non-communicable illnesses and is characterized by alteration of glucose metabolism and vascular and neural damage. According to differences in the pathogenic mechanisms, DM is divided into type I and type II (Fan W. et al., 2022). It has been demonstrated that immunocytes and autoimmune antibodies-triggered destruction of pancreatic β-cells and concomitant insulin insufficiency are deeply associated with the onset of type I DM (Renard, 2022). Previous studies have determined the importance of insulin resistance in the development of type II DM. Specifically, inflammatory factors decrease the activities of insulin receptor in hepatocytes, muscle cells, and adipocytes, accompanied by insulin receptor substance-induced intracellular signaling transduction inhibition, glucose transporter generation and translocation suppression, and glucose uptake and metabolism disruption (Passarelli and Machado, 2021; Xiao et al., 2022). A hyperglycemic environment facilitates the occurrence of inflammation and oxidative stress, which exacerbate vascular and myocardial complications and enhance the risks of diabetic disability and mortality (Lu et al., 2021; Semwal et al., 2021; Yu et al., 2022). There is evidence showing that KD administration effectively decreased blood glucose content, elevated circulating insulin level, prevented weight gain, and improved glucose tolerance in streptozotocin-induced hyperglycemic rats, without causing toxicity. The in-depth mechanism exploration suggested that anti-diabetic roles of KD could be ascribed to alleviation of oxidative stress-evoked pancreatic β-cells injury, as seen in the percentage increase in intact β-cells in the islets of Langerhans with denser insulin, serum content increment of SOD and level decrement of MDA (Zhang et al., 2007). These findings indicated that KD was capable of alleviating the progression of type I DM *via* suppression innate immune cells-induced impairments on pancreatic β-cells. In addition, KD failed to raise the level of circulating insulin in normal healthy rats, suggesting that KD was less likely to induce hypoglycemic reactions seen with sulfonylureas and glinides, further implying the safety profile of this natural phytochemical. The findings of Budluang et al. (2017) showed that aqueous extracts of *Anoectochilus burmannicus*, an *Anoectochilus* species different from *A. roxburghii*, were capable of suppressing insulin resistance of adipocytes by disrupting TNF-α-activated inflammation cascades. This result, along with the fact that pro-inflammatory cytokines contributed to the onset of insulin insensitivity *via* weakening the generation of functional proteins like insulin receptor and glucose transporter 4 participating in glucose uptake and transport, hinted that KD probably protected against the development of type II DM. Protein tyrosine phosphatase 1B (PTP1B), an inhibitor of insulin signaling transduction *in vivo*, was found to have regulatory roles in inflammation development (Wu J. et al., 2021). Owing that KD acted as a negative mediator of PTP1B activity, it was possible that KD produced hypoglycemic effects by abrogating PTP1B activation in an anti-inflammation-dependent manner (Wang et al., 2015).

**FIGURE 3:**
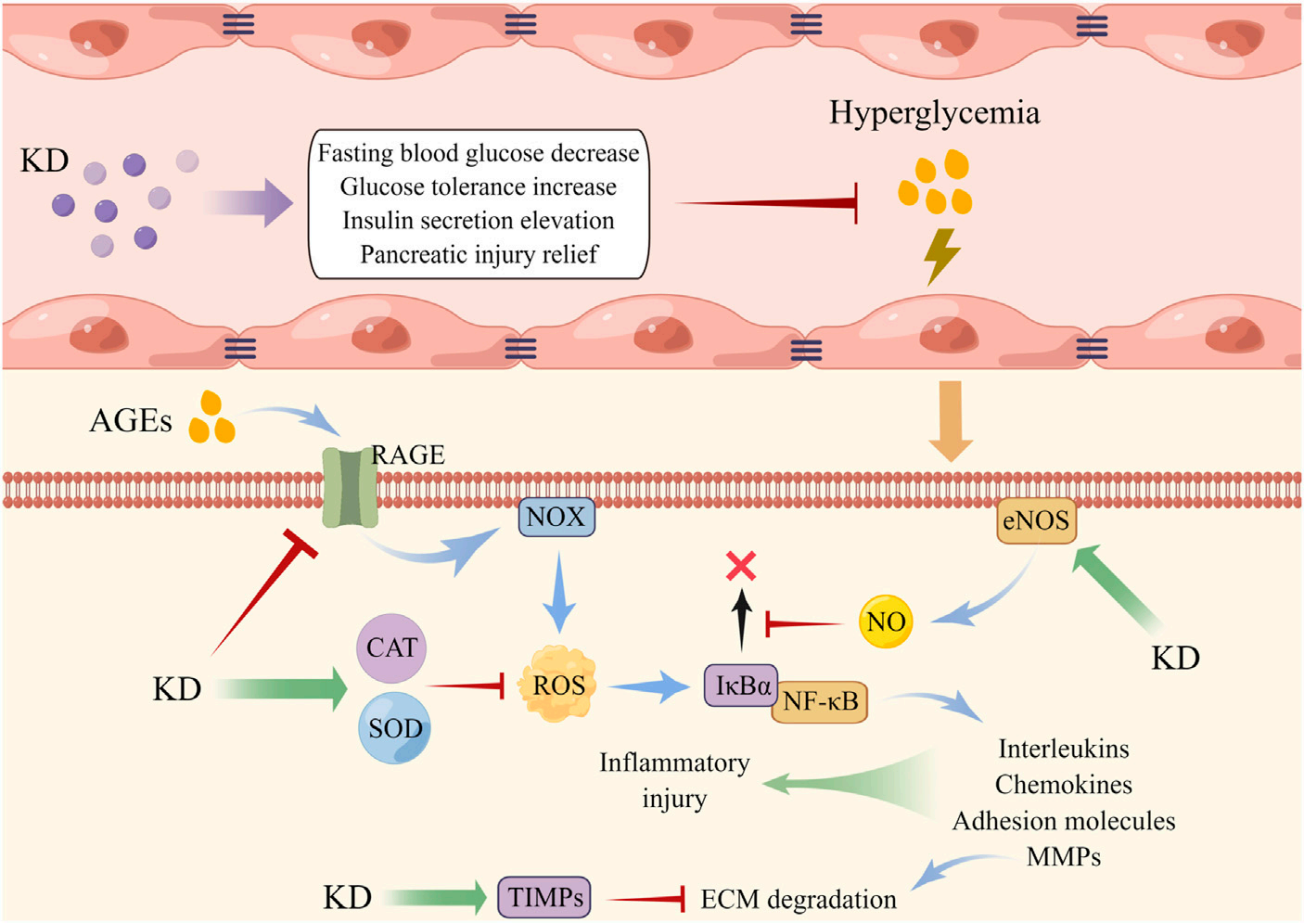
KD administration alleviated endothelial dysfunction triggered by hyperglycemia. AGEs displayed an increased level under the environment of high glucose, which then induced intracellular NOX activation and ROS overload, accompanied by NF-κB activation and expression of inflammatory factors. KD elevated the contents of antioxidants and NO to disrupt the interaction of ROS with NF-κB, thereby weakening endothelial injury.

Once irritated by hyperglycemia, cellular oxidases become overactive and antioxidants are down-regulated, both of which induce vast ROS accumulation and provoke oxidative impairment of intracellular macromolecules, thereby resulting in endothelial dysfunction (Peng et al., 2021; Wautier and Wautier, 2022). Moreover, ROS overload is found to trigger the activation of cytosolic NF-κB and sequential translocation into the nucleus for transcribing pro-inflammatory factors like interleukins and MMPs, further damaging the lumen of vasculature. The disordered conditions of endothelial cells (Ecs) induced by oxidative stress and inflammation elements cause vasoconstriction, platelet activation and aggregation, vasopermeability increase, and lipoprotein deposition and are viewed as the central pathogenic component of macro- and micro-vascular diabetic complications (Shi and Vanhoutte, 2017; Chen et al., 2020; Luk et al., 2022). Liu and collaborators found that oral administration of KD decreased the content of blood glucose and improved collagen sedimentation and fibrosis of the vessel wall. They also discovered that the level of decline of SOD, CAT, NO, and TIMP1/2 and of the ascent of NF-κB-MMP2/9 axis in Ecs evoked by high glucose was reversed by KD pre-conditioning, indicating that it ameliorated hyperglycemia-elicited vascular malfunction through mechanisms dependent on ROS clearance and inflammation abrogation (Liu et al., 2013). Advanced glycation end products (AGEs), a class of by-products synthesized from non-enzymatic glycosylation reactions of excessive sugar with lipid and/or protein, have high concentrations in the circulation of diabetic models and have been illustrated to exhibit pivotal roles in vascular dysfunction. The mechanism of this is hypothesized to be through binding with the transmembrane receptor (RAGE) of Ecs, followed by NOX activation and ROS generation, which in turn diminishes NO availability, activate the NF-κB pathway and facilitate the expression of downstream pro-inflammatory factors including adhesion molecules and chemokines, ultimately amplifying inflammation response and weakening endothelial survival (Jing et al., 2021; Liu Y. et al., 2022). It has been documented that depressed NO content and limited cellular viability and elevated levels of RAGE, ROS, NF-κB, monocyte chemoattractant protein 1 (MCP-1), and intercellular cell adhesion molecule 1 were observed in AGEs-insulted Ecs, which was restored by KD post-treatment, demonstrating that antagonizing AGEs/RAGE/ROS-elicited activation of NF-κB is possibly associated with the vasoprotective capabilities of KD in diabetic injury (Liu et al., 2016) (Figure 3).

### Skeletal and articular damage

**FIGURE 4:**
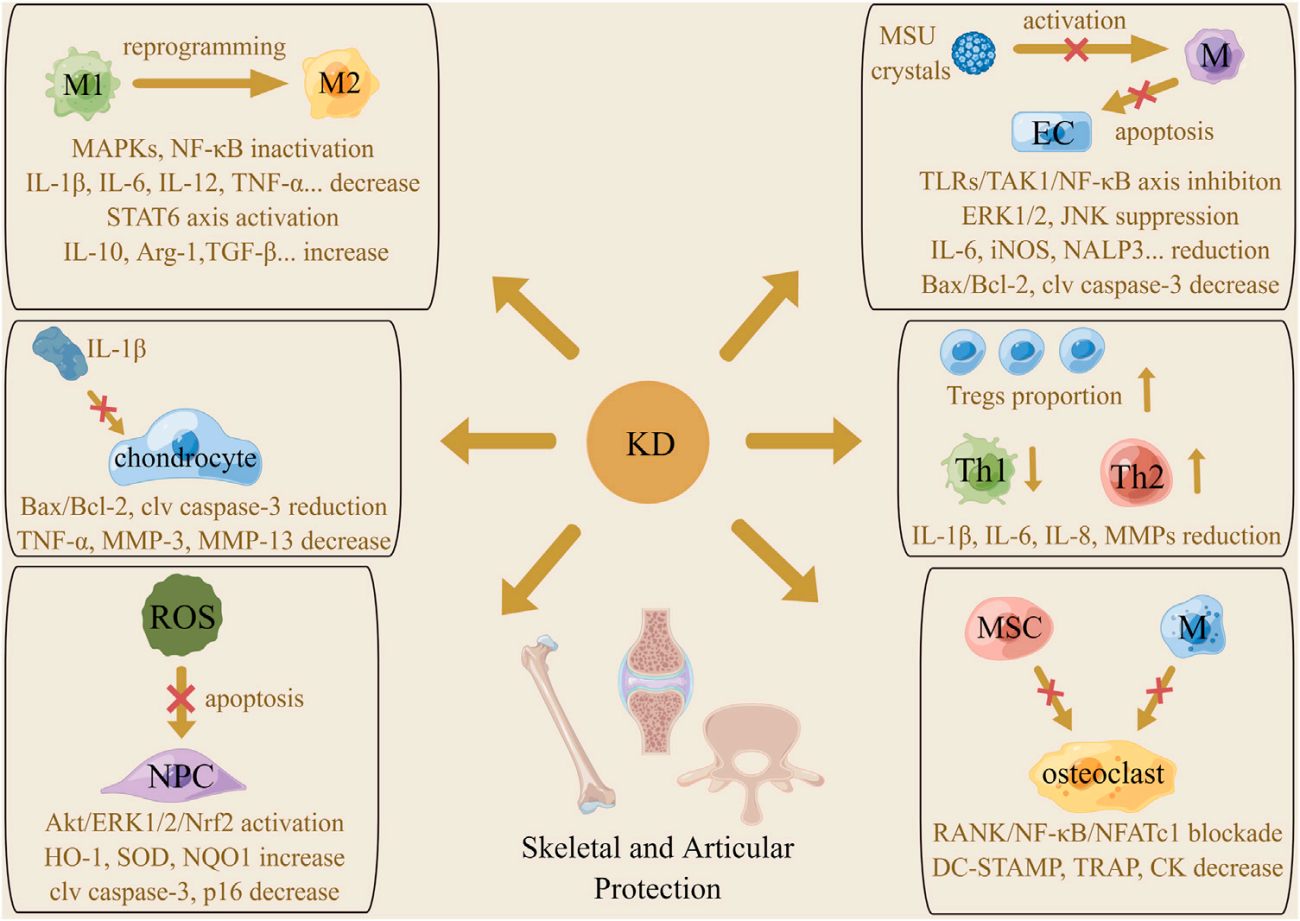
The mechanism of the actions underlying protective effects of KD on the skeletal and articular system. Macrophages phenotype reprogramming, proportion change of T cell subtypes, amelioration of chondrocytes and ECs apoptosis and inhibition of pro-inflammatory cytokines generation were involved in KD-related beneficial roles in arthritis management. Promotion of ROS scavenging and abolishment of osteoclast transdifferentiation were associated with the therapeutic action of KD on intervertebral disc degeneration and osteoporosis.

The primary histopathological alterations of osteoarthritis (OA) include articular cartilage loss, endochondral ossification, subchondral bone sclerosis, and synovial inflammation (Terkawi et al., 2022; Sun Z. et al., 2022). Emerging evidence supports that macrophages are a critical target in OA therapy, in light of their substantial heterogeneity and plasticity properties (Karlinsey et al., 2022). Typically stimulated by signals from the TLRs axis activated by LPS, IFN-γ and TNF-α, resting macrophages are polarized to the pro-inflammatory M1 phenotype with the high expression of hallmark CD86 and iNOS and produce inflammatory cytokines like IL-1β, IL-6, IL-12, and TNF-α, called the classical macrophage activation. It has been reported that activated M1 macrophages not only release inflammatory factors to induce tissue damage but also produce killing effects on microbes, parasites, and tumors (Eshghjoo et al., 2022). On the other hand, the induction of the interleukins comprising IL-4, IL-10, and IL-13 shifts macrophages to the anti-inflammatory M2 phenotype featured by expression of IL-10, TGF-β1, and VEGF and onset of immunosuppressive reactions, which is called the alternatively macrophage activation (Louiselle et al., 2021). According to their stimuli, M2 macrophages are divided into four subtypes, which share some overlapping features, including suppressing the functions of pro-inflammatory cytokines and up-regulating the expression of hallmark arginase 1 (Arg-1) and CD206. It has been documented that M2 macrophages often appear in the microenvironment of damaged sites to induce tissue regeneration, angiogenesis, and remodeling. Moreover, these cells are reported to exist in tumor tissues to promote immune escape of tumor cells by exerting immunosuppressive effects (Sun et al., 2020). Zhou et al. (2019) reported that LPS and IFN-γ triggered the M1-type switching of macrophages as expected, but KD addition reduced the level of IL-6, IL-1β, TNF-α, IL-12, and iNOS/Arg-1 and initiated the reprogramming of M1 macrophage to M2 phenotype. The underlying mechanism was hypothesized to be the repression of the IKK/NF-κB, ERK1/2, JNK, and p38 cascade. Furthermore, KD was found to directly enhance macrophage shifting towards the M2 subtype by activating the STAT6 axis, characterized by an increase in Arg-1, IL-10, CD206, and PGC-1β. Laboratory detection found that KD administration under IL-1β stimulation resulted in an inhibition of signaling transduction of mitochondrial-dependent apoptotic pathways and a decrease in pro-inflammatory factors like IL-6, TNF-α, and ECM-degrading enzymes like MMP-3 and MMP-13 chondrocytes. Zhou and colleagues postulated that KD might be a breakthrough of OA therapy, as KD was able to protect chondrocytes from inflammatory cytokines-induced apoptosis and reduce cartilage-destructive substances release by strengthening M1 subtype reprogramming and M2 subtype polarization. Indeed, *in vivo* experiments witnessed the diminution of anterior cruciate ligament transection-evoked expression of pro-apoptotic and inflammatory factors, reduction of the ratio of M1/M2 macrophages, increase of proteoglycan content and articular cartilage thickness, and alleviation of inflammation permeation in the synovium after KD intervention (Figure 4).

Commonly affecting the first metatarsophalangeal joint, monosodium urate (MSU) crystals deposit in large joints including the knee, wrist and ankle, causing fairly inconveniences for individuals with gouty arthritis (GA) (Desai et al., 2017; Cabau et al., 2020). Assembling in synovial tissues and fluids, MSU crystals are combined with immunoglobulins and complement proteins to recruit macrophages to the target site. MSU crystals interact with TLR2/4 on the membrane of these leukocytes and are phagocytosed using CD14 as an adaptor molecule, accompanied by activation of downstream pro-inflammatory pathways and generation of inflammation effectors responsible for joint injury (Papanagnou et al., 2016; Galozzi et al., 2021). By promoting chemokine secretion, engaging NALP3 inflammasome, and inducing production of membranolysis-related DAMPs, MSU crystals are capable of enhancing neutrophils arrival, interleukins maturation, and inflammation signaling to exacerbate synovial damage. Assaulted by inflammatory cytokines, ECs of the inner layer of vessels at an inflamed location undergo apoptotic processes participating in vascular dysfunction, which is another mechanism by which MSU crystals evoke the expansion of articular erosive lesions (Cronstein and Sunkureddi, 2013; Makkar et al., 2020). This is in agreement with Han et al. (2016), who found that MSU stimulation dramatically increased inflammatory infiltration of the synovial membrane, ankle swelling, and tail-flick response of rats, while KD treatment effectively alleviated these signs and symptoms by regulating inflammation in macrophages and endothelial apoptosis. Concretely speaking, KD pre-conditioning inhibited MSU-induced level elevation of IL-1β, IL-6, TNF-α, iNOS, COX-2, and NALP3 in macrophages by abrogating the intermediate IKK kinase activity and blocking the signal flow of TNFR-associated factor 6 (TRAF6)/TGF-β-activated kinase 1/NF-κB axis and JNK and ERK1/2 pathway. These actions thereby weakened ECs impairment caused by conditioned medium from MSU-treated macrophages, as determined by western blotting which showed raised Bcl-2 and decreased Bax and cleaved caspase-3 in the KD cohort. For purpose of controlling the start of sterile inflammation, KD was found to inhibit MSU crystals phagocytosis by macrophages *via* repressing the expression of CD14 and TLR2 on the cytomembrane upon MSU insult.

The formation of autoantibodies called rheumatoid factors (RFs) is the key culprit contributing to onset of Rheumatoid arthritis (RA) (Li and Zhang, 2020). Once discerned and entrapped by antigen presenting cells (APCs), the RFs are delivered to lymph nodes for activating T and B cells which synthesize inflammatory substances and induce synovitis and joint injury. Evidence from recent studies has identified T_regs_ as a safeguard favoring RA extinction, due to their potent abilities for inflammation relief (Smolen et al., 2016; Lin et al., 2020). Mice with RA, established by intradermal immunization with type II collagen, displayed paw edema and swelling, osteoclast and macrophage accumulation, synovial hyperplasia, and cartilage and bone destruction of the knee joint. Oral administration of KD decreased the expression of IL-1β, TNF-α, MMP-9, and CD68 and increased the level of IL-10 in joint tissues, followed by improvement of joint injury, RA incidence, and mean severity score. Mechanistic analyses depicted that KD up-regulated T_regs_ proportion to display immunosuppressive effects on Th1 and B cells, thus inhibiting the generation of inflammation factors as opposed to directly modulating Th1/Th2 differentiation (Hsiao et al., 2016). Additionally, among the local cell populations involved in RA progression, fibroblast-like synoviocytes are the dominant category producing bioactive substances to induce synovial thickening, chondrocyte death, and ECM loss (Smolen et al., 2016). There was evidence that KD intervention had inhibitory roles in the proliferation rate and expression of IL-1β, IL-6, IL-8, MMP-1, MMP-3, and MMP-13 of MH7A cells exposed to TNF-α, further highlighting the curative potential of KD from the perspective of inflammation restraint (Xu et al., 2022).

When osteoclast-induced bone absorption outweighs osteoblast-triggered bone formation, skeletal homeostasis is disordered, and osteoporosis occurs (Gkastaris et al., 2020). Scholars have reported certain factors susceptible to osteoporosis, including inflammatory stimuli, estrogen insufficiency, lack of exercise, poor nutritional status, glucocorticoid abuse, and cigarette smoking (Wang et al., 2017; Martiniakova et al., 2020). Specifically, inflammatory stimuli not only restrict the polarization of mesenchymal stem cells (MSCs) towards osteoblasts but also promote the osteoclastogenic activities and reinforce the biological functions of osteoclasts, signifying the beneficial effects of agents that promote anti-inflammatory behaviors to ameliorate osteoporosis (Lacativa and de Farias, 2010; Wu D. et al., 2021). As shown by Hsiao and co-workers, the decline of trabecular thickness, bone volume and trabeculae number, and ascent of trabecular separation in the distal femoral metaphysis, rise of plasma C-terminal telopeptide of type I collagen concentration and ALP activity and overall osteoclast percentage and released cytokines were all seen in ovariectomized mice. KD treatment improved these osteoporosis-related pathological alterations by compromising inflammation pathway-mediated differentiation, fusion and activation of osteoclasts (Hsiao et al., 2013). By restraining the activity of IKK, KD interrupts the signal transmission from receptor activator of NF-κB (RANK)/TRAF6 to downstream NF-κB/nuclear factor of activated T-cell, hampering macrophage colony-stimulating factor. and RANK ligand-provoked switching of MSCs and macrophages to osteoclasts and limiting the generation of DC-specific transmembrane protein, tartrate-resistant acid phosphatase, MMP-9, and creatine kinase which were implicated in bone resorption performances.

In addition to its effects of the inflammation-induced ECM devastation, the phenotype transformation of nucleus pulposus (NP) cells, and the conversion of the gel-like, highly hydrated NP to an unorganized fibrous substance, oxidative stress is a critical pathogenic factor accelerating intervertebral disc degeneration (IDD) development (Risbud and Shapiro, 2014; Feng et al., 2017; Cao et al., 2022). Normally, the electron transport chain in the mitochondria plays a modulatory role in energy storage, while its malfunction can trigger excessive ROS accumulation, followed by the loss of NP cells and disc degeneration (Saberi et al., 2021). In static surroundings, nuclear factor erythroid 2-related factor 2 (Nrf2) is retained in the cytosol *via* coupling with kelch-like ECH-associated protein 1. Once activated, Nrf2 is released from the dimer and translocates to the nucleus for binding to the promoter of antioxidative genes and enhancing their transcription (Zhou J. et al., 2022). Inactivation of Nrf2 and the subsequent inhibition of antioxidants expression are a further mechanism required for ROS-elicited NP damage (Hu et al., 2019). After KD administration in mice with IDD, there is a dramatic amelioration of the loss of T2-weighted signal in punctured discs, collapsed disc height, destruction of matrix, and disappeared boundary between annulus fibrosus and NP. *In vitro* investigations have illustrated that KD abated mitochondrial membrane potential and permeabilization to mitigate the dysfunctional extent and strengthened Akt/ERK1/2-induced activation of Nrf2 to increase the synthesis of HO-1, SOD and NQO1 in NP cells insulted by oxidative stress, which suppresses ROS generation and facilitated ROS clearance and then weakened oxidative damage-related cellular apoptosis and senescence (Wang et al., 2019). Considering that overproduction of ROS is pathogenic in many bone and joint diseases, regardless of IDD, exploring the involvement of antioxidative abilities of KD in its protective effects against these disorders will lay a solid foundation for the use of KD in future clinical practice.

### Age-related macular degeneration

Age-related macular degeneration (AMD) is a common vision-threatening illness that is a leading cause of blindness in the elderly, with the prevalence projected to rise as the population ages (Mitchell et al., 2018). AMD is classified into early, intermediate, and late phases, featured by incipient drusen deposits and focal hypopigmentation, progressively loss of the retina and retinal pigment epithelium (RPE), choroidal neovascularization and neurosensory retina disassembly, and advanced appearance of permanent visual impairment (Al-Zamil and Yassin, 2017; Gil-Martinez et al., 2020). The risk factors for AMD include gender, age, smoking, nutrition, body-mass index, diet, and certain environmental stimuli. Among the core events contributing to AMD, oxidative stress and inflammatory response are considered to be pivotal etiologies, as they damage the RPE and promote friable neo-angiogenesis (Kauppinen et al., 2016; Flores et al., 2021). A prior study demonstrated that H_2_O_2_ stimulation arouses the apoptosis enhancement of RPE cells, which is rescued by KD co-incubation, as exemplified by a decrease in the Bax level and an increase in the Bcl-2 content. The ROS-eliminating behavior of KD is probably linked to its protective actions on cellular viability (Luo X. et al., 2018). Simultaneously, KD dose-dependently inhibits the neovascularization of HUVECs irritated by conditional medium from damaged RPE cells, hypothetically through prohibiting pro-angiogenic VEGF synthesis by inactivating inflammation cascades of ERK1/2, p38 and NF-κB.

### LPS-induced impairment

When LPS is discerned by TLRs on the membrane of macrophages, it undergoes cell-mediated internalization, and its intracellular pro-inflammatory pathways become activated with cytokines production, resulting in tissue damage (Belkaid and Hand, 2014). Macrophages release chemokines to induce neutrophils aggregation and act as APCs to further activate lymphocytes, which results in the generation of plentiful cytotoxic factors and occurrence of inflammation-related sequelae, such as disseminated intravascular coagulation and endotoxin shock (Braza et al., 2016; Fitzgerald and Kagan, 2020). The findings of Hsiao et al. (2011) declared that KD treatment significantly alleviated murine symptoms of endotoxic shock including diarrhea, ruffled fur, and ocular exudates and raised the survival rate of mice provoked by LPS. The authors supported that these protective actions of KD were due to inhibition of the nuclear translocation of NF-κB and its transcriptional activity and simultaneous increase in the level of suppressor of cytokine signaling 3, which led to an expression reduction of IL-1β, TNF-α, NO, MCP-1, and macrophage migration inhibitory factor and to a content elevation of IL-10, showing promising potential for mitigating cytokine storm. It cannot be ignored that multiple pathophysiological processes contribute to LPS-induced organ impairments, such as cellular apoptosis, vascular dysfunction, oxidative stress, interstitial fibrosis, and platelet aggregation. Among these, oxidative stress is identified as the most closely linked event other than the inflammatory reaction. There is evidence that mitochondrial homeostasis deprivation, ROS accumulation, NOX activation, increased MDA level, and lowered SOD activity are present in the pulmonary tissue of mice challenged with LPS, despite the elevated contents of IL-1β, TNF-α, IL-6 and high-mobility group box 1 (Yang Y. et al., 2022). However, KD efficaciously rescues the viability reduction of lung epithelial cells damaged by LPS *in vitro* and protects the lung from LPS-induced pathological injury *in vivo*. This might be due to activation of the AMP-activated protein kinase (AMPK)/Nrf2 cascade by KD that facilitates the dynamic balance of mitochondrion shifting from a fission to a fusion stage, followed by mitigation of mitochondrial injury and diminution of ROS burden, thereby resulting in downstream inhibition of inflammatory signaling and cellular apoptosis.

### Lipid metabolism dysfunction

Evidence has documented that dyslipidemia exerts contributing roles in development of diverse diseases including atherosclerosis, hepatitis, pancreatitis, diabetes and malignancies (Zhang et al., 2007; van Diepen et al., 2013; Gao et al., 2021). Hepatocytes and adipocytes, the principal regulators controlling lipid droplets anabolism and catabolism, are verified to be the main sites targeted by medications aimed to manage dyslipidemia. A number of studies have supported the versatile lipid-lowering abilities of KD, as exemplified by decreasing blood total cholesterol and triglyceride (TG) content, reducing hepatocellular TG level and enhancing activity of adipocyte-related lipolysis (Du et al., 2001; Liu et al., 2013; Cheng et al., 2015; Lee et al., 2019). Cumulative evidence indicates that pro-inflammatory stimuli had the capacity of inducing fatty acid and cholesterol biosynthesis, TG lipolysis, and very low-density lipoprotein and free fatty acid secretion while suppressing fatty acid oxidation and cholesterol excretion in cell types responsible for lipid metabolism (Khovidhunkit et al., 2004; Glass and Olefsky, 2012). This fact, along with the observation that KD is capable of disrupting the crosstalk between lipid and glucose metabolism and inflammatory signaling in DCs and lymphocytes and mediating the activity of the energy sensor AMPK during the execution of inflammation clearance in ALD model, suggests that KD treatment might perturb the transduction of inflammatory pathways to affect the activation of AMPK, thus indirectly activating lipid-scavenging pathways and invalidating lipid-producing cascades, which remains to be further elucidated (Xiang et al., 2016; Gao et al., 2021).

## Cultivation, isolation and synthesis of KD

The genus *Anoectochilus* (Orchidaceae family) contains at least 40 species that grow in the evergreen broadleaved or bamboo forests of southern regions of East Asia and the west Pacific islands. Of these, *A. roxburghii*, the most famous indigenous herbal plant employed in folk remedy, is renowned as “King Medicine” or “Jewel Orchid,” owing to its broad-spectrum curative roles in antagonizing multiple diseases. Although *A. roxburghii*-related patented drugs have been developed, such as Jinxianlian Capsule and Oral Liquid, ecological habitat loss, relentless collection, animal consumption, and low budding and growth rates suggest that there is an extinction tendency existing in the wild resource of this medicinal plant (Wang et al., 2011; Ye et al., 2017; Wu et al., 2020). Therefore, artificial cultivation and harvest techniques have been launched as substitutes to boost yield and meet market demand.

Endophytes owned beneficial effects on the host plant by accelerating germination and growth, enhancing tolerance to stresses and promoting secondary metabolite generation. Coculturing *A. roxburghii* with endophytic fungi strains J162 and J211 and F-23 fungus resulted in an increase in the biomass and active metabolites accumulation of the *A. roxburghii* (Zhang et al., 2013; Ye et al., 2020). Moreover, as the environment of post-harvest exhibits important functions that influence the commercial values of botanic materials, researchers are investigating the packaging methods and storage parameters for the preservation of the active compounds of *A. roxburghii* (Wei et al., 2022). Notably, both environmental protection and appropriate exploitation are fundamental to maintaining the species propagation of *A. roxburghii* for continued pharmaceutical and nutritional use.

With the advancement of chromatography approaches, multiple chemical components of the whole plant of *A. roxburghii* have been discovered, including polysaccharides, flavonoids, glycosides, organic acids, alkaloids, steroids, nucleosides, and triterpenes (Ye et al., 2017). Ethnopharmacological experiments have demonstrated that KD (3-(R)-3-β-D-glucopyranosyloxybutanolide) is the dominant bioactive constituent underlying the therapeutic actions of *A. roxburghii*, due to its potent ability to repress inflammation and oxidative stress (Qi et al., 2018). Considering its limited availability and shortage of plant materials, several strategies have been carried out to increase KD production to meet medical demand. At first, tissue culture approaches were used to maximize the propagation of the species and the deposition of metabolite KD under specific conditions (Du et al., 2000). Later, Jin et al. (2017) used plant cell culture technology and found that rhizomes of *A. roxburghii* could serve as alternative raw materials when fostered within the bioreactor system and could result in increased accumulation of KD. Afterwards, by optimizing the proportion of the nutritional components and additive usage, scholars successfully elevated the biomass of rhizomes and induced the mass production of KD (Luo W.-Y. et al., 2018; Jin et al., 2018). With respect to the factors influencing extraction efficiency, multiple lines of evidence disclose that the efficiency of KD extraction depends on the drug phase, solution category, extraction method, reaction temperature, and other operational parameters (Yuan et al., 2022). Wei and colleagues found that using water as the solvent and freeze drying are commonly coupled with the high extraction rate that was superior to ethanol solvent and hot-air drying. Compared to supercritical fluid, heated reflux, Soxhlet. and microwave-assisted extraction, the ultrasound assisted extraction possessed favorable advantages for efficient KD production, owing to time-saving, cost-effective, eco-friendly, ease of operation, and high repeatability properties (Wei et al., 2020). Furthermore, the response surface methodology had been employed to maximize the output of KD from raw materials by optimizing solvent dosage and reaction condition during the extraction processes (Yang et al., 2020). In consideration of the high price of natural *A. roxburghii* extraction, artificial chemical synthesis is viewed as a promising avenue to offset the provision scarcity of KD. Suzuki et al. (2005) first reported the efficient large-scale synthesis of KD by construction of the aglycon residue after the region- and stereo-selective glycosylation. Through the glycoxidation of the benzyl protected D-glucose with (R)- or (s)-4-hyfroxy-tetrahyfrofuran-2-one, the synthetic procedures became more concise (Zhang et al., 2005). Moreover, using a chemo-enzymatic approach with fewer reaction steps and improved experimental conditions, the efficiency of synthesizing KD was significantly enhanced (Zhang et al., 2014).

## Pharmacokinetic profiles of KD

It has been found that the liquid phase of drugs absorbed in the bloodstream is a prerequisite for pharmacokinetic analysis. Owing to its chemical structure of having a lactone group in the aglycone ring, KD is susceptible to hydrolysis and shows high *ex vivo* instability (Qi et al., 2018). To obtain as accurate metabolic data as possible for KD *in vivo* as truly, multiple attempts have been made to enhance the drug’s stability, sensitivity, and specificity by adjusting ambient parameters, altering co-reagents composition, and updating testing methods. For instance, modulating the response temperature, pH, and processing time and utilization of acetic acid, the stability of KD in rat plasma (*in vitro*) was markedly increased (Rehman et al., 2016b). Then with the novel HILIC-MS/MS, pharmacokinetic indicators of KD were determined, which uncovered its features of rapid assimilation and clearance (T_1/2_: 0.68 ± 0.1 h, T_max_: 0.03 h, C_max_: 1225.9 ± 72.6 ng/ml) and adequate tissue distribution (Vz: 19.9 ± 3.4 L/kg). Similarly, utilizing improved sample preparations and advanced bioanalytical approaches, Zhang et al. (2018) effectively and reliably acquired statistics reflecting the absorption, distribution, elimination, and bioavailability of KD, as presented by pharmacokinetic results. By possessing diverse converting factors like cytochrome P450 enzymes and flavin monooxygenase, the liver acts as the central site determining the activation, detoxification, degradation, and excretion of drugs. Studies have explored the metabolic stability of KD by employing the liver microsomal screening system, which illustrated that the amount of the drug remaining was 74.2% and 77.8% after incubation of this drug with rat and human liver microsomes for 2 h separately, suggesting that KD might be stable *in vivo*, despite the metabolic loss caused by hepatocytes, and exhibits decent bioavailability to produce therapeutic effects (Rehman et al., 2015). Furthermore, it was observed that KD did not influence the enzymatic activities of CYP isozymes, and interestingly, when used in combination with lovastatin and amlodipine, it exerted negligible actions on the transformation of these drugs, clarifying potent safety and efficacy of KD, even in combination therapy (Rehman et al., 2016a).

## Challenges and trends

The favorable effects of KD on alleviating inflammatory and oxidative damages have been confirmed, but several concerns should be tackled before it is applied in clinical practice. First, the weakness of undersupply and the cost of raw materials deserve to be emphasized, as artificial synthesis and alternative source materials are likely to be feasible solutions. Moreover, although limited experiments have found the pharmacokinetic profiles of KD, the gap between effective dose and toxic dose *in vivo* is unknown, and dose toxicity testing performed on large primates is warranted. As a kind of hydrophilic phytochemical, KD possesses improper properties, including burst release, low retention, and rapid elimination when used *in vivo* (Mirchandani et al., 2021). Hydrogels are 3-dimensional porous biomaterials made up of cross-linked polymer chains. A series of substances forming polymers have been discovered, such as natural chitosan, hyaluronic acid, and alginate and synthetic poly (ethylene glycol) and poly (vinyl alcohol) (Sonker et al., 2021). Due to its excellent biocompatibility, biodegradability, low immunogenicity, high safety, and controlled release, hydrogels have been used as drug delivery vector to increase the stability and bioavailability of drugs (Mirchandani et al., 2021). According to previous studies, the design of rational hydrogel carriers with immunoregulatory abilities is determined by physicochemical parameters of biomaterials, including dimensionality, cross-linking, stiffness, niche pH, surface charge, topography, thermal conductivity, swelling rate, and molecular weight (Bu et al., 2022). It could be speculated that the KD encapsulated in hydrogels possesses superior efficiency in inhibiting inflammatory response and oxidative injury. Furthermore, the current treatment modalities of KD lack the requisite targeting functions, regardless of *via* intravenous infusion, intraperitoneal injection, or gastric perfusion. Binding KD with targeting peptides and encapsulating it into targeting exosomes or nanoparticles are promising engineering strategies for enhancing the therapeutic accuracy, reducing application dosage and ameliorating off-target effect. It has been found that metabolism reprogramming is intimately implicated in regulation of immune reactions, elucidating that KD-induced alterations on metabolite profiles and their interactions with inflammation pathways are rewarding for unraveling the novel molecular mechanisms responsible for anti-inflammatory effects of KD (Møller et al., 2022). There is evidence that inflammatory responses exert bidirectional roles in different stages of several diseases, particularly tissue injury. When tissue is damaged, local neutrophils are activated to phagocytize invading pathogens, dead cells, and tissue debris, and they release pro-inflammatory cytokines to recruit various immunocytes in the target sites to accelerate the cleanup procedures. The inflammation reactions peak within 24–48 h and fade away at about 7 days post-injury (Maruyama et al., 2020). Once entered into the repair phase, accumulation of inflammatory factors is detrimental to the processes of cell proliferation and tissue regeneration. Thus, the dynamic balance between the duration of pathogens and debris clearance and the initiation of tissue repair determines the advantageous or disadvantageous properties of inflammation responses, implying that selection of intervention time point is the key challenge for KD application. Identifying the precise actions and durations of inflammation responses on different stages of disease development will be beneficial for guiding the utilization of KD. In addition, the hydrogel carries with drug release ability under the control of photo-thermal or other specific conditions are feasible options.

## Conclusion

**FIGURE 5:**
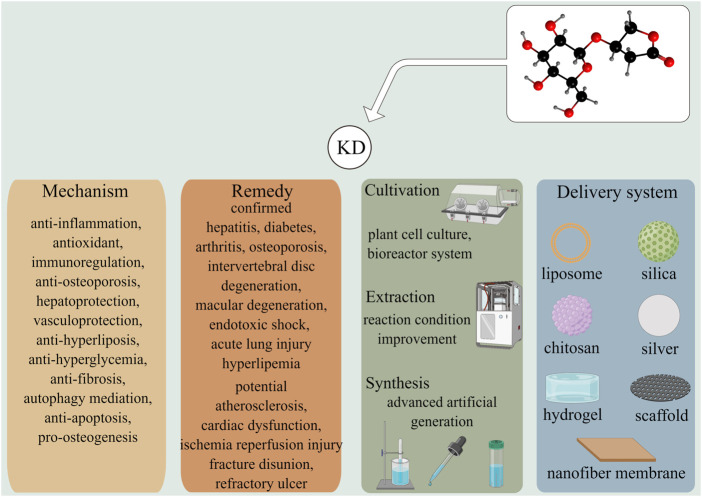
KD exhibits effective roles in prohibiting the development of several diseases including hepatitis, diabetes, arthritis, osteoporosis, and lung damage *via* versatile molecular mechanisms. Other illnesses, such as atherosclerosis, fracture disunion, and refractory ulcer were had potentials to be treated by KD, which were worthy to be further elucidated. The improvements in the cultivation, extraction and synthesis techniques were likely to overcome the deficiency of KD-producing raw materials and met the demands of medical care. Multiple drug delivery systems had been developed to enhance the stability, bioavailability, and safety of phytochemicals *in vivo*.

A vast array of research has ascertained the pleiotropic pharmacological effects of KD. By counteracting inflammation signaling and oxidative stress, KD is demonstrated to suppress the occurrence and development of multiple diseases, including hepatitis, diabetes, arthritis, osteoporosis, macular degeneration, and acute lung injury, without inducing adverse events (Figure 5). It is notable that findings from recent studies have outlined the anti-bacterial and anti-biofilm abilities of KD (Fan M.-Z. et al., 2022). These functions, together with the promotive roles of inflammatory and oxidative damages in the pathogenesis of chronic ailments, manifest KD’s immense potential for use in refractory traumatic disorders, especially bone non-union in fracture repair and diabetic dermal ulcers. With the advancement in biomolecular technology, delivery systems have been developed with the intention of optimizing the pharmacokinetic profiles of drugs by encapsulating them in specific materials. Taking into account the quick clearance and low bioavailability of KD, it is promising to enhance compound persistence, stability, and availability to reduce toxicity through material engineering. Since the large majority of the studies concerning therapeutic application of KD have been laboratory-based research, double-blind, large sample, multi-center, and randomized controlled trials are advocated to lay a solid foundation for the translational utilization of KD in future clinical practice.
